# Rinsing of allograft bone does not improve implant fixation: A study in 12 dogs

**DOI:** 10.3109/17453674.2013.797314

**Published:** 2013-05-31

**Authors:** Jeppe Barckman, Jorgen Baas, Mette Sørensen, Joan E Bechtold, Kjeld Soballe

**Affiliations:** ^1^Orthopaedic Research Laboratory, Aarhus University Hospital, Denmark; ^2^Orthopaedic Biomechanics Laboratory, Excelen and Minneapolis Medical Research Foundations, University of Minnesota, Minneapolis, MN, USA.

## Abstract

**Background and purpose:**

Impacted morselized allograft bone is a well-established method for reconstructing bone defects at revision surgery. However, the incorporation of bone graft is not always complete, and a substantial volume of fibrous tissue has been found around grafted implants. We hypothesized that rinsing the bone graft may improve graft incorporation by removing the majority of immunogenic factors present in blood, marrow, and fat.

**Methods:**

We implanted a cylindrical (10- × 6-mm) porous-coated Ti implant into each proximal tibia of 12 dogs. The implants were surrounded by a 2.5-mm gap into which morselized fresh frozen allograft bone was impacted. The bone graft was either (1) untreated or (2) rinsed in 37°C saline for 3 × 1 min. After 4 weeks, the animals were killed and implant fixation was evaluated by mechanical push-out and histomorphometry.

**Results:**

The groups (rinsed vs. control) were similar regarding mechanical implant fixation (mean (SD)): shear strength (MPa) 2.7 (1.0) vs. 2.9 (1.2), stiffness (MPa/mm) 15 (6.7) vs. 15 (5.6), and energy absorption (kJ/m^2^) 0.5 (0.2) vs. 0.6 (0.4), The same was evident for the new bone formation on the implant surface and around the implant: ongrowth (%) 6 vs. 7 and ingrowth (%) 9 vs. 9. Although not statistically significant, a 61% reduction in fibrous tissue ongrowth and 50% reduction in ingrowth were found in the rinsed group.

**Interpretation:**

Within the limits of this experimental model, we did not detect any benefits of rinsing morselized allograft bone prior to impaction grafting.

Bone graft impaction is one well-established method of reconstructing bone defects at prosthetic revision surgery. This method enables restoration of the bone-bed and optimizes the initial mechanical support of the revision prosthesis (Burchardt 1983, Gie et al. 1993, Schreurs et al. 1998, Toms et al. 2004). Among bone graft options, autologous bone graft is considered to be the gold standard, but limited volume and donor-site morbidity makes its use less favorable in revision surgery. Allograft bone is readily available and is the most commonly used graft material. However, it has been shown that incorporation of allograft bone into the host bone is not always complete (Linder 2000, Van der Donk et al. 2003), and considerable formation of fibrous tissue in the bone implant interface has been demonstrated clinically (Heekin et al. 1995, Nelissen et al. 1995). This may be a contributory factor in the higher failure rates and poorer functional outcome in patients following revision surgery (Espehaug et al. 1998, Overgaard 2011).

In contrast to autogenic bone, allogeneic bone graft may induce an extended immunogenic response, leading to increased formation of fibrous tissue (Burchardt 1983). Most antigenic cells of allograft bone are found in the bone marrow (Czitrom et al. 1985). By removing blood, marrow, and fat from the bone graft, improved bone graft incorporation has been demonstrated in a bone chamber model in goats (Van der Donk et al. 2003). Furthermore, rinsing has been shown in vitro to physically improve the mechanical stability of impacted morselized allograft bone (Ullmark 2000, Dunlop et al. 2003, Arts et al. 2006). The authors concluded that the removal of fat, marrow, and soft tissue optimized the contact between the bone chips. This allows better particle interlock and tighter graft compaction, and thereby improves the mechanical stability of the bone graft.

To our knowledge, the effect of rinsing allograft bone prior to impaction around an implant in vivo has not been tested. From the results of Dunlop et al. (2003) and Van der Donk et al. (2003), one would expect improved mechanical implant fixation and improved osseointegration of the implant after rinsing. We therefore hypothesized that rinsing of the allograft bone would improve implant fixation and enhance incorporation of bone graft, as evaluated by mechanical push-out and histomorphometry.

## Material and methods

**Figure 1.**
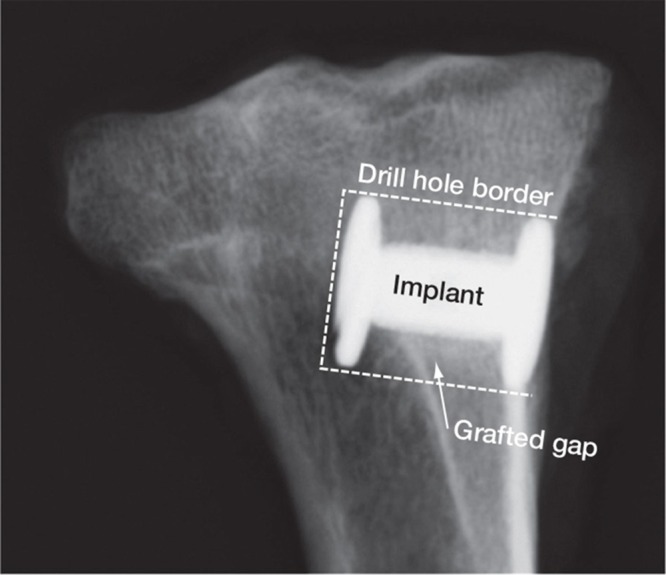
Gap-implant in the proximal tibia. Radiograph taken post mortem.

The experiment was conducted as a paired, randomized, controlled animal study on 12 dogs. Sample size was determined before the experiment and was based on previous studies using the same allograft implant model, assuming normally distributed data and a coefficient of variance of the paired difference of 30%. The minimal clinically relevant difference to be detected in this study was set to 30%. Two-sided α and β were set to 5% and 20%, respectively. The animals received one porous-coated titanium implant in the metaphyseal cancellous bone of each proximal tibia (Figure 1). All implants were surrounded by a nominal 2.5-mm concentric gap into which either rinsed or untreated allograft bone was impacted. The implantation site (left or right limb) of each treatment group was alternated systematically with random start. The observation period was 4 weeks.

The animal experiment was approved by the Institutional Animal Care and Use Committee (Minneapolis Medical Research Foundation (MMRF), Minneapolis, MN, submission ID 10-11-02). Surgery and observation were performed at the animal care facilities of MMRF, according to the regulations of the National Institutes of Health. Preparation of the bone samples, mechanical testing, and histomorphometry were carried out at the Orthopaedic Research Laboratory of Aarhus University Hospital, Denmark.

### Animals

12 skeletally mature female American hounds with a mean weight of 23 (SD 2.0) kg and a mean age of 47 (SD 21) months were included in the study.

### Implants

**Figure 2.**
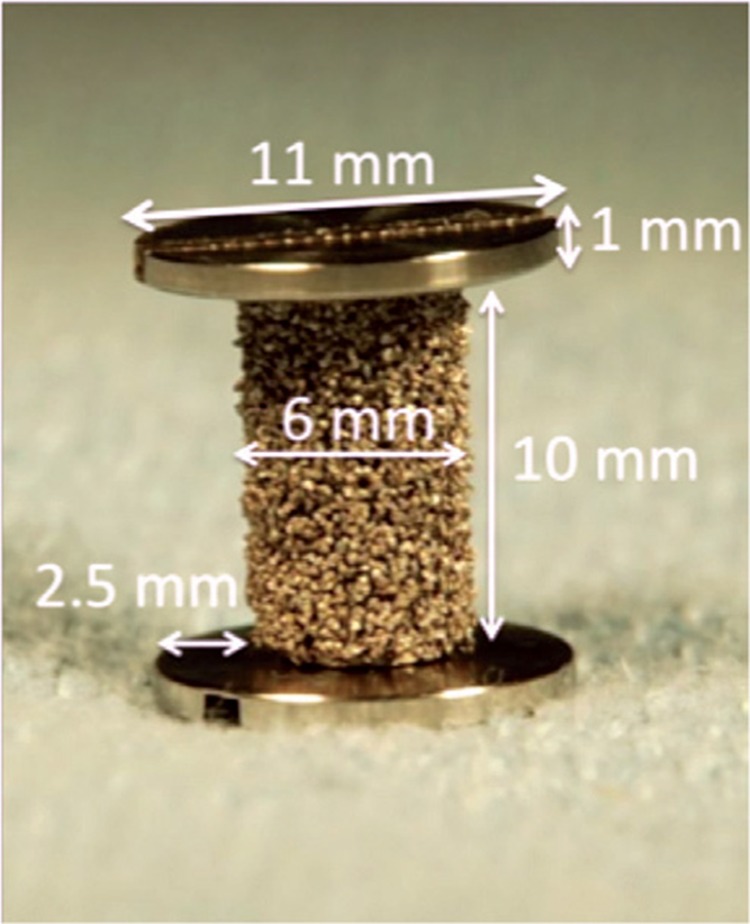
Experimental porous-coated titanium 2.5 mm gap implant with measurements.

For the experiment, we used 24 porous-coated titanium alloy (Ti-6A1-4V) implants manufactured by DePuy Inc. (Warsaw, IN). The implants were custom-made and cylindrical, with a height of 10 mm and a mean diameter of 5.93 (SD 0.09) mm. Before autoclave-sterilization, the implants were cleaned for 10 min in an ultrasonic bath of trichloroethylene with final baths in alcohol. An end-plate with a diameter of 11 mm was attached to one end of the implant and inserted into an 11-mm pre-drilled bone defect. The end-plate centered the implant, providing a uniform circumferential 2.5-mm peri-implant gap. After impaction grafting, an 11-mm top screw was mounted at the outer end of the implant, ensuring implant stability and bone graft containment (Figure 2).

### Bone graft

Bone graft was harvested immediately post mortem, under sterile conditions, from 2 dogs not included in the study and stored at –80°C. The proximal humerus, the proximal tibia, and the distal femur were used. Before preparation, the bones were thawed and all soft tissue and cartilage was removed. The bones were morselized using a standard bone mill (Biomet, Warsaw, IN) on fine setting, creating bone chips of 1-3 mm in largest diameter. The bone chips from the 2 dogs were mixed together.

### Bone graft rinse

**Figure 3.**
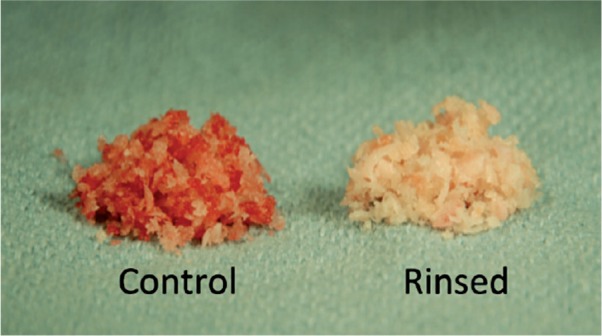
Bone graft of the 2 treatment groups.

The gap volume of this model is 0.67 cm^3^. From previous studies performed by the same surgeon on this model, we knew that approximately 1.38 g of morselized bone can be impacted into this gap (Barckman et al. 2013). 12 samples and 4 reserve portions of bone allograft were needed in each treatment group. Therefore, under sterile conditions, 2 portions of morselized allograft bone each weighing 22 g (1.38 g × 16) were placed in separate glass containers. In the intervention group, 220 mL of 37°C saline (10 mL saline per 1 g bone graft) was added and gentle digital stirring was performed. After 1 min, the water was poured off and the bone graft was gently squeezed between the fingers. This procedure was repeated 3 times. Finally, the rinsing water was poured through a fine sieve and there was no visible bone remaining. The volume of the rinsed bone graft was measured in 10 mL syringes, using maximum manual force by the same surgeon. This procedure squeezed the water from the bone graft. An identical procedure was conducted to measure the volume of the unrinsed control bone graft (Table 1). The bone graft in each group (Figure 3) was divided into 16 equal portions and stored in sterile double-containers at –80°C. The preparation of the bone graft for the whole study was undertaken in one session on the day before surgery commenced.

**Table 1.** Allograft wash

	Weight	Volume	Volume/impl.
	(g)	(cm^3^)	(cm^3^)
Control	22.08	19.62	1.23
Rinsed	22.44	17.82	1.11
Difference rinsed/control	1.63%	–9.17%	

Volume and weight were measured after water was drained with maximum manual pressure.

### Surgery

Under general anesthesia and under sterile conditions, a skin incision was made on the anteromedial part of the tibia just distal to the joint line. The periosteum was elevated. A 2.5-mm Kirschner wire was inserted perpendicular to the anterior medial plane of the proximal tibial surface and 12 mm distal to the tibiofemoral joint line. A hole of 12 mm in depth and 11 mm in diameter was drilled with a cannulated drill bit at a maximum speed of 2 rotations per second, and the edges of the drill hole were trimmed with a scalpel. An end-plate was attached to one end of the implant and inserted into the drill hole with a specially designed hollow cylinder impaction tool. The same tool, which fits into the defect between the implant and the border of the drill hole, was used to manually impact the bone graft into the gap. To ensure homogeneous distribution of the graft in the defect, one third of the graft was impacted into the bottom third of the gap, then one third of the graft was impacted into the middle third of the gap, and finally the remaining graft was impacted into the most superficial third of the gap. To ensure uniform implant placement and minimal variance in impaction technique, all surgeries were undertaken by the same surgeon (JBA). Finally, an end-plate was mounted onto the implant to maintain its concentric location within the gap, and the soft tissues including the periosteum were closed in layers. Bupivacaine (0.5%) was administered locally following closure of the skin. A 1-g dose of Ceftriaxone was given intravenously to each animal before surgery. Postoperatively, the dogs were given Ceftriaxone (1 g) intramuscularly for 3 days. A Fentanyl transdermal patch (75 μg/h) lasting 3 days was given for postoperative analgesia. The animals were allowed unlimited activity.

After the observation period of 4 weeks, the dogs received sedation with Acepromazine (0.5 mg/kg), they were anesthetized using Propofol (4 mg/kg), and were killed with an overdose of hypersaturated barbiturate.

### Bone-implant specimen preparation

The proximal tibias were dissected free and immediately frozen and stored at –80°C. Preparation of the specimens was performed blind. Before dividing the bone into the mechanical and histomorphometric sections, the proximal tibias were thawed and the outermost 1 mm of the implant-bone sample below the end-plate was cut off and discarded. The remaining 9 mm of implant with surrounding bone was divided into 2 sections perpendicular to the long axis of the implant, using a water-cooled diamond band saw (Exakt; Exakt Apparatebau, Norderstedt, Germany). The inner 5.5-mm section of the implant was processed for histomorphometric evaluation by dehydration in graded ethanol (70–100%) containing 0.4% basic fuchsin, and embedded in methylmethacrylate (Art. 800590; Merck, Darmstadt, Germany). The outer 3.5-mm section was stored at –20°C and used for mechanical push-out testing.

Using vertical uniform random (VUR) sectioning technique (Baddeley et al. 1986), four 30-µm-thick central histological sections were cut parallel to the axis of the implant with a hard-tissue microtome (KDG-95; MeProTech, Heerhugowaard, the Netherlands). These techniques provide highly reliable results with negligible bias (Baas 2008). Finally, the sections were surface-stained with 2% light green (Light Green SF; BDH Laboratory Supplies, Poole, UK) for 2 min, rinsed and mounted on glass. This preparation provided red staining for non-calcified tissue and green staining for calcified tissue. The different types of calcified tissues such as newly formed bone (woven bone) and bone graft (lamellar bone) were categorized on the basis of their morphological characteristics (Gotfredsen et al. 1989).

### Mechanical testing

The thawed specimens were tested to failure by axial push-out test on an MTS Bionics testing machine (MTS 858 Mini Bionix) with MTS Test Star 790.00 software version 4.0C. The testing was performed blind and in one session. The specimens were placed with the cortical side facing upwards on a metal support jig, with the 6-mm-diameter implant centered over a 7.4-mm opening and under a cylindrical test probe of 5 mm diameter. A preload of 2 N defined the contact position at the start of the test. The implant was pushed from the surrounding bone in the direction of the implant axis at a velocity of 5 mm/min. Data on load (N) vs. implant displacement (mm) were continuously recorded for every 10 µm of implant displacement. To normalize for differences in thickness and diameter of the implants in the push-out specimens, the load data were converted to strength by approximation of the implant surface area (implant diameter × height × π). The shear strength (in MPa) was determined from the load applied until failure of the bone-implant interface (represented as the fist peak on the load-displacement curve) and calculated as shear strength = load / πDL where D is the average diameter and L is the height of the implant. Shear stiffness (in MPa/mm) was obtained from the linear section of the load-displacement curve and calculated as shear stiffness = (Δload / πDL) / ΔT, where T is the displacement. The energy absorption (in kJ/m^2^) was calculated as the area under the strength-displacement curve until failure (Søballe 1993).

### Histomorphometric analysis

**Figure 4.**
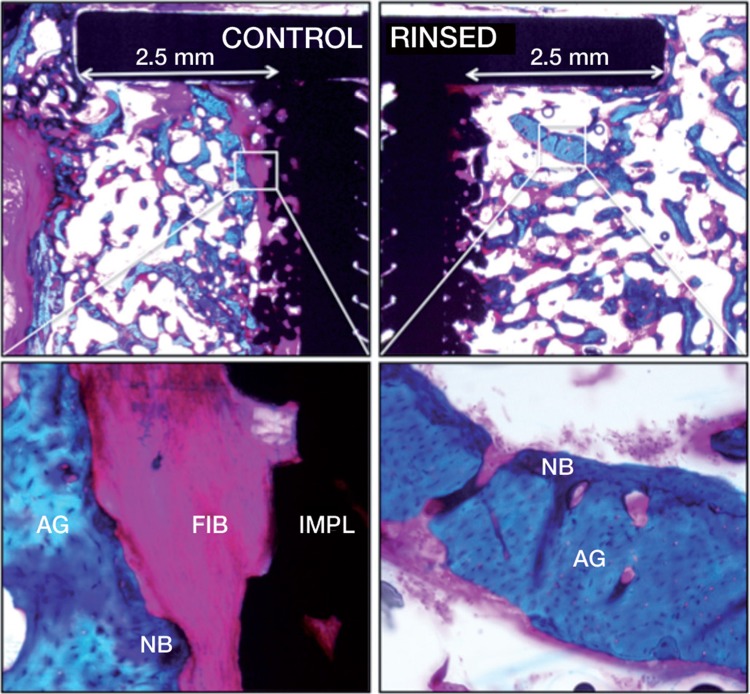
Histology: representative histological sections from the two groups. The sections shown are from the two implants inserted in the same animal. Porous-coated Ti implants are surrounded by a 2.5-mm concentric defect. The sections are cut parallel to the long axis of the implant. Left-hand panels (control): fibrous (FIB) tissue at the implant (IMPL) surface. Right-hand panels (rinsing): new bone (NB) on graft (AG) remnants.

We performed blind quantitative histomorphometry using stereological software (newCAST version 3.4.1.0; Visiopharm A/S, Horsholm, Denmark). The region of interest was defined as the area from the implant surface extending 2.0 mm into the circumferential gap. Volume fractions of new bone, allograft bone, and fibrous tissue were quantified by point-counting technique (Gundersen et al. 1988). Tissue ongrowth was defined as tissue directly at the implant surface and was estimated by line intercept technique (Baddeley et al. 1986). The 4 vertical sections representative of each implant were analyzed and sampled (Figure 4). The mean surface intercept count for each implant was 1,378 (SD 176) and the mean gap volume point count for each implant was 3,551 (SD 483).

### Statistics

All mechanical data followed a normal distribution, as evaluated by QQ plots of the residuals. Difference between the 2 groups was evaluated with paired t-test. The mechanical data are presented as mean (SD) and the absolute differences between treatment groups as mean with 95% confidence intervals (95% CIs). Data from the histomorphometrical assessment were normally distributed for the parameters new bone and allograft bone, as evaluated by residual QQ plots of the paired difference. The calculated estimates are presented as mean (SD) and the absolute differences between treatment groups are presented as mean (95% CIs). The area fraction and volume fraction of fibrous tissue were not normally distributed, mainly due to many values close to zero. Wilcoxon signed-rank test was used, and estimates are given as median and interquartile range (IQR). Differences between medians or means were considered statistically significant for p-values less than 0.05. The statistical analyses were performed using STATA statistical software (version 11.2).

## Results

All 12 animals were fully weight bearing within 3 days of surgery and they completed the 4-week observation period without clinical signs of infection or other complications. All 24 implants were available for mechanical and histomorphometrical analysis.

### Mechanical testing

We found no statistically significant difference in mechanical fixation of the implants (as defined by ultimate shear strength, apparent shear stiffness, or total energy absorption) in the group with rinsed allograft bone compared to the group with untreated allograft bone (Table 2). From the calculated estimates and the 95% confidence intervals, the difference between groups regarding ultimate shear strength was found to be between 22% improvement and 11% worsening. For the apparent shear stiffness, it was between 19% improvement and 19% worsening and for energy absorption it was between 40% improvement and 40% worsening.

**Table 2.** Biomechanical results

Groups	Shear strength	Shear stiffness	Energy absorption
	(MPa)	(MPa/mm)	(KJ/m^2^)
Control **^a^**	2.7 (1.0)	15 (6.7)	0.5 (0.2)
Rinsed **^a^**	2.9 (1.2)	15 (5.6)	0.6 (0.4)
Rinsed – Control **^b^**	0.2 (–0.3 to 0.6)	0.0 (–2.8 to 2.8)	0.1 (–0.2 to 0.3)
p-value (t-test)	0.5	1.0	0.5

**^a^** Mean (SD). **^b^** Absolute difference between treatment groups. Mean (95% CI).

### Histomorphometry

Compared to the untreated allograft controls, the intervention group showed no statistically significant difference in any parameters regarding area fraction of tissue ongrowth or volume fraction in the gap around the implant (Table 3). When comparing the rinsed group to the control group, the difference regarding new bone surface coverage was found to be between 50% increase and 18% decrease, new bone volume fraction was found to be between 33% increase and 23% decrease, and bone graft volume fraction was found to be between 21% increase and 12% decrease. Regarding fibrous tissue, a 61% reduction in fibrous tissue ongrowth and 50% reduction in ingrowth were found but, due to large variance, this difference was not statistically significant.

**Table 3.** Histomorphometrical analysis: tissue area fraction on implant surface and tissue volume fraction in the gap (ingrowth)

	New bone	Bone graft	Fibrous tissue
	mean% (SD)	mean% (SD)	median% (IQR)
Implant surface			
Control	6 (3.7)	0 (0.2)	18 (3–31)
Rinsed	7 (2.9)	0 (0.2)	7 (1–18)
Rinsed – Control **^a^**	0 (–1.1 to 3.0)	0 (–0.1 to 0.3)	
p-value	0.3 **^b^**	0.4 **^b^**	0.2 **^c^**
Ingrowth			
Control	9 (2.7)	13 (4.1)	4 (1–6)
Rinsed	9 (3.3)	13 (4.4)	2 (1–5)
Rinsed – Control **^a^**	0 (–2.1 to 3.0)	0 (–1.5 to 2.7)	
p-value	0.7 **^b^**	0.5 **^b^**	0.3 **^c^**

IQR: interquartile range. **^a^** Absolute difference between treatment groups. Mean (95% CI). **^b^** T-test **^c^** Wilcoxon

## Discussion

Our findings did not confirm our hypothesis; we found no statistically significant difference in the histomorphometrical evaluation or the mechanical evaluation between the 2 groups. The study was powered to detect relative differences of 30% between the groups. The assumptions for this were largely met. The uncertainty of the estimated differences based on the 95% CIs was somewhat higher (Table 3), especially for newly formed bone on the implant surface—which may have been as much as 50% higher to 18% lower in the rinsed group than in the untreated control group.

Our experimental implant model is simple and has a high degree of variance control. However, the implants are inserted extra-articularly and are therefore not influenced by direct load or joint fluid. Furthermore, the surgery was conducted in the bones of young healthy animals and not in compromised bone that often surrounds a loose implant in humans. The observation period of 4 weeks was chosen based on previous experience with graft resorption and new bone formation in the same animal model (Baas 2008), and a paired study design was used to allow implants impacted with rinsed or unrinsed allograft bone to be compared in the same animal. The detectable clinically relevant threshold was set at 30%, and the choice was based on the consideration that bone quality in revision surgery is highly variable; an experimental study must therefore show a substantial effect before being clinically relevant.

The design of our study regarding graft volume also had limitations. Equal volumes of untreated morselized allograft bone were measured in both groups before the rinsing procedure. The 9% volume reduction seen in the intervention group (Table 1) resulted in a mean impacted volume in the control group of 1.23 cm^3^ as compared to 1.11 cm^3^ in the intervention group. We did not find any bone tissue in the rinsing water, and we therefore consider the volume reduction to be mainly caused by the removal of blood, fat, and bone marrow tissue. Thus, the impacted volumes of bone graft were considered to be the same in both groups, although the actual volume of impacted tissue was different. This design enabled us to isolate the effect on osseointegration of reducing the immunogenic factors. An alternative study design would be to impact an equal volume of graft material irrespective of of how it was prepared. If so, the total volume of bone graft would have been larger in the intervention groups due to less filling of blood, fat, and marrow tissue (Voor et al. 2008), which would possibly improve early implant fixation due to denser bone graft impaction (Ullmark 2000, Dunlop et al. 2003, Arts et al. 2006). This may have been a more clinical approach, but the results would have been more difficult to interpret because both reduction of the immunogenic load and denser impaction would have been influencing the fixation of the implant.

We were unable to detect any statistically significant difference between the 2 groups. One explanation for the similar results in the 2 groups could be that the presence of immunogenic factors does not matter. When comparing the incorporation of fresh autograft bone and fresh allograft bone, the initial inflammatory process is comparable (Burchardt 1983). Both graft types illicit an inflammatory response involving formation of a hematoma, and recruitment and activation of immune cells, which results in release of osteoinductive factors such as cytokines, growth factors, and prostaglandins for stimulation of bone regeneration (Frost 1989, Khan et al. 2005). Rinsing may lower the overall immunogenic load, but this might not influence the bone regeneration.

Another possible explanation would be that the presence of immunogenic factors does matter but that our rinsing method was insufficient. Different methods have been used for rinsing of the bone graft, including pulse-lavage washing (Hirn 2001, Dunlop et al. 2003, Haimi et al. 2008, Ibrahim et al. 2012) and manual rinsing (Voor et al. 2008). The manual rinsing method we used was chosen because of its simplicity and the possibility of preserving a broad range of bone graft particle sizes, which has been shown to be important for optimal mechanical strength of the impacted bone graft (Brewster et al. 1999). In contrast, rinsing by pulse-lavage may cause smaller bone graft particles to be lost, thus narrowing the particle-size distribution. By our method, the bone grafts were cleared macroscopically of obvious fat, blood, and marrow tissue (Figure 3), as was the case in other studies (Van der Donk et al. 2003, Kwong et al. 2005). The most antigenic cells of the bone graft are in the bone marrow (Burchardt 1983, Czitrom et al. 1985) and although our rinsed bone graft appeared clean, this may have been insufficient in terms of reducing foreign-body reaction. It could be that we only removed the erythrocytes, leaving the majority of the immunogenic factors. We cannot confirm or deny this possibility.

We purposely chose a combination of defect size and observation time for which we would expect bridging of the bone-grafted defect with newly formed bone (Søballe et al. 1992). The purpose of the model was to perform a comparative analysis of treatments at the time point at which this had occurred, by comparing parameters of biomechanical implant fixation and histomorphometric analysis of the tissues on the implant surface and in within the grafted defect. We find an observation time of 4 weeks relevant, as this is the time point of potential transient weakening of the bone-implant interface. This is due to weakening of the bone graft, caused by bone resorption, whereas the newly formed bone is still immature and weak pending remodeling (Jeppsson et al. 2003). Furthermore, this is the time point where we think early subsidence may be more frequently expected to occur, which in clinical studies is an early predictor of later loosening (Kärrholm et al. 1994).

After 4 weeks, the grafted defect of the model was bridged by newly formed bone in both groups. Any observations on the difference in speed with which the new bone formation progressed through the defect were therefore not possible. Although this was not the subject of this experiment, it could be interesting and relevant to our understanding of the effects of reducing the load of foreign epitopes in the allograft. This may be better understood using other models such as the bone conduction chamber developed by Aspenberg et al. (1993).
